# Agreement of stall‐side and laboratory major crossmatch tests with the reference standard method in horses

**DOI:** 10.1111/jvim.15710

**Published:** 2020-02-04

**Authors:** Melissa S. Fenn, Araba D. Bortsie‐Aryee, Gillian A. Perkins, Sabine Mann, Joy E. Tomlinson, Emma M. Wood, Susan E. Mix, Tracy Stokol

**Affiliations:** ^1^ Cornell University Department of Clinical Sciences, College of Veterinary Medicine Ithaca New York; ^2^ Cornell University Department of Population Medicine and Diagnostic Sciences, College of Veterinary Medicine Ithaca New York; ^3^ Cornell University Baker Institute for Animal Health, College of Veterinary Medicine Ithaca New York

**Keywords:** alloantibody, agglutination, blood incompatibility, blood type, point‐of‐care test, transfusion reaction

## Abstract

**Background:**

Crossmatching is used to prevent life‐threatening transfusion reactions in horses. Laboratory methods are laborious and technically challenging, which is impractical during emergencies.

**Hypothesis/Objectives:**

Evaluate agreement between a stall‐side crossmatch kit (KIT) and a laboratory method (LAB) in horses with known and unknown blood types.

**Animals:**

Twenty‐four blood‐typed and alloantibody‐screened healthy adult horses (Aim 1) and 156 adult horses of unknown blood type (Aim 2).

**Methods:**

Prospective, blinded study. Expected positive (n = 35) and negative (n = 36) crossmatches among 24 antibody and blood‐typed horses were used to determine sensitivity and specificity of KIT and LAB against the reference method. Agreement in 156 untyped horses was evaluated by reciprocal crossmatch (n = 156).

**Results:**

Sensitivity (95% confidence interval [CI]) for LAB and KIT compared with expected reactions was 77.1% (59.9%‐90.0%) and 91.4% (77.0%‐98.2%), and specificity 77.8% (60.9%‐89.9%) and 73.5% (55.6%‐87.1%), respectively. The KIT was 100% sensitive for Aa reactions; LAB was 100% sensitive for Qab; and both were 100% sensitive for Ca. Cohen's κ agreement for LAB and KIT with expected positive and negative reactions (n = 71) was moderate (0.55 [0.36‐0.74]) and substantial (0.65 [0.47‐0.82]), respectively. Agreement was fair comparing LAB with KIT in Aim 1 (0.30 [0.08‐0.52]) and in untyped horses in Aim 2 (0.26 [0.11‐0.41]).

**Conclusions and Clinical Importance:**

Agreement between KIT and LAB with expected reactions was blood type dependent. Performance of both methods depends on blood type prevalence.

**Abbreviations**
GEL: gel column agglutination method
KIT: stall‐side gel crossmatch kit
LAB: laboratory crossmatch procedure
RCB: red blood cell

## 1 INTRODUCTION

Whole blood transfusion is a vital tool in equine critical care, the purpose of which is restoring oxygen delivery to tissues in patients with severe anemia. Transfusions are not necessarily benign, and reactions vary in severity from mild hives to anaphylaxis and death.^1^ Knowledge about the donor's blood type and presence of antibodies in the recipient can help prevent adverse reactions. Blood groups are defined by inherited antigens on the red blood cell (RBC) surface. They contribute to recognition of self and can elicit antibody production when introduced into the circulation of an animal with RBCs lacking that antigen. This becomes clinically relevant during blood transfusions, where allogeneic incompatibilities affect patient safety.^2^

There are 8 RBC groups in horses (A, C, D, K, P, Q, U, and T). Each group corresponds to a particular gene for which ≥2 alleles exist. These genes produce surface molecules known as factors, with >30 different factors identified. Consequently, there are over 400,000 possible equine blood types.^3^ Approximately 90% of horses have no naturally‐occurring alloantibodies. Of the 10% that do, anti‐Aa and anti‐Ca antibodies occur most often.^4^ Anti‐Ca antibodies have minimal clinical effects, instead, anti‐Aa and Qa are highly immunogenic and associated with severe reactions.^4^, ^5^

Pre‐transfusion testing is indicated to minimize the risk of incompatible transfusions.^5^, ^6^ Blood typing and crossmatching should be performed before transfusion to identify an appropriate donor, but this is challenging in emergency situations. Only a few laboratories perform blood typing in horses. Crossmatching is more readily available as a bench‐top laboratory assay, but it is time consuming and requires technical expertise. Therefore, most emergency transfusions are performed without compatibility testing.

A new stall‐side crossmatch kit has been developed for horses and has yet to be made available commercially. It uses a gel column agglutination (GEL) method that has been used for crossmatching in dogs.^7^ A previous study compared different crossmatch methods in horses,^6^ but did not evaluate the particular stall‐side kit used in our study. The kit is offered as an efficient way to establish transfusion compatibility. It can be run at all hours by lay individuals, providing results in <20 minutes. Therefore, if deemed a sensitive and specific method, it would allow for safe blood transfusions in emergency situations.

Our objective was to evaluate agreement between this commercial gel‐based stall‐side crossmatch kit (KIT) and the standard laboratory method (LAB) in horses of known and unknown blood types. Aim 1 compared sensitivity, specificity, and agreement for the KIT and LAB in crossmatch reactions with expected outcomes based on blood type and antibody screening. Aim 1 also determined if method agreement depended on blood type. Aim 2 compared agreement between KIT and LAB in a large population of horses of unknown blood types. This approach mimicked field situations where unscreened horses may be transfused with untyped blood. We hypothesized that the KIT would be a sensitive and specific crossmatch method and would have good agreement with the LAB method in horses of known and unknown blood types.

## 2 MATERIAL AND METHODS

Blood (20 mL) was collected from the jugular vein of horses into no‐additive vacutainer tubes and vacutainer tubes containing K‐ethylenediaminetetraacetic acid (EDTA) for blood typing and crossmatch testing by the KIT and LAB methods. As a blinded study, the personnel sampling the horses and identifying crossmatch combinations were different than those performing the crossmatches. In addition, the person performing the KIT method was blinded to the results of the LAB crossmatch, and vice versa, until the end of the study.

All protocols were approved by Cornell University's Institutional Animal Care and Use Committee, and horses were housed in accordance with federal guidelines for the humane care and use of laboratory animals.

### 2.1 Aim 1

Twenty‐one adult clinically healthy horses housed at Cornell University's Equine Park were used in this prospective methods comparison study between July 2017 and June 2018. Additional anti‐sera was kindly donated from the University of California, Davis Hematology Laboratory (anti‐Qab, n = 1) or identified in‐house from incompatible crossmatches performed at Cornell University's Clinical Pathology Laboratory on samples submitted to the Animal Health Diagnostic Center for routine neonatal isoerythrolysis testing (anti‐Aa, n = 1; anti‐Ca, n = 1; May 2017). Prior transfusion history was unknown and it also was not known if any horse had been diagnosed with neonatal isoerythrolysis as a foal or had produced foals that developed neonatal isoerythrolysis.

#### 2.1.1 Reference standard method (expected reactions)

Serum and anticoagulated whole blood from Aim 1 horses were submitted for blood typing (for blood groups A, C, D, K, P, Q, and U) and screening for anti‐RBC hemolytic and agglutinating antibodies (against Aa, Ab, Ac, Ad, Af, Ca, Da, Dg, Dk, Ka, Pa, Pb, Pc, Qa, Qb, Qc, Ua, and donkey factor) to the University of California‐Davis Veterinary Medical Teaching Hospital Hematology Laboratory. Eleven of the 21 horses were blood typed in February 2017 and 10 in January 2018. This laboratory uses a herd of typed horses, previously described standard antisera and macroscopic tube crossmatch methods for determining antibody profiles and blood types.^8^ Briefly, screening for antibodies was performed by incubating serial dilutions of a serum sample with a series of equine RBC of known blood types. This procedure was repeated with the addition of complement for the hemolysin assay. The presence of agglutination and hemolysis was assessed visually, and antibodies were reported as present or absent. If antibodies were detected but could not be further identified (ie, if it could not be determined which RBC antigens they were directed against), they were classified as “unidentified anti‐RBC antibodies.”^9^

Based on the blood types and antibody screens, expected positive and negative crossmatch reactions were set up by 1 investigator (T. Stokol) using different donor‐recipient pairs to achieve approximately even numbers of expected positive (n = 35) and negative (n = 36) crossmatches. An example of how expected positive and negative crossmatches were determined is as follows: A recipient with anti‐Qabc alloantibodies matched against a donor with Qa antigen on its RBC would be an expected positive (ie, incompatible reaction), but the same recipient against a donor with Aa antigen on its RBC would be an expected negative (ie, compatible reaction). Recipients with unidentified anti‐RBC antibodies were placed in an additional “unknown” crossmatch group (n = 19) because we could not know if the donor had the corresponding antigen. Crossmatches using horses with only anti‐donkey antibodies as recipients were considered negative reactions.

### 2.2 Aim 2

Horses of unknown blood types and antibody profiles were used in this prospective study between September 2017 and August 2018. Horses were enrolled at convenience from Cornell's Equine Park (excluding the 21 already blood typed), Cornell University's equestrian and polo teams, and privately owned horses. Written client consent for privately owned horses was obtained before blood sampling. Eligibility criteria for enrollment included healthy horses >6 months old, with health being determined by physical examination.

### 2.3 Crossmatch methods

A trained veterinarian, undergraduate student, or medical technologist in the Clinical Pathology Laboratory performed the LAB crossmatches (S.E. Mix, A.D. Bortsie‐Aryee, E.M. Wood) and a single veterinarian from Cornell University Hospital for Animals performed all the KIT crossmatches (M.S. Fenn). Results for both methods were verified by independent blinded observers, another medical technologist for LAB and another veterinarian for KIT. A third person was consulted if there were discrepant results within method. All crossmatches were performed within 12 hours of blood collection except for the 3 sources of anti‐sera (Aim 1), which were stored frozen at −80°C. To maintain blinding, additional serum from typed and antibody‐screened horses was stored similarly frozen. Frozen sera were thawed in a warm water bath at 37°C before use.

For both methods, LAB and KIT, reactions of 1‐3+ (details below) were considered positive or an incompatible crossmatch, with 0 equivalent to no agglutination and a compatible crossmatch.

#### 2.3.1 Laboratory crossmatch procedure

This test was performed using the standard macroscopic and microscopic agglutination and hemolysis method^8^, ^10^ used for routine crossmatches in the Clinical Pathology Laboratory. In this assay, no‐additive tubes were centrifuged at 3800*g* for 5 minutes to harvest serum. EDTA‐blood from the “donor” was centrifuged at 1000*g* for 1 minute and washed 3 times in phosphate‐buffered saline, creating a final 2% suspension of RBCs. “Recipient” serum (fresh or frozen‐thawed) was diluted 1:2 in 0.9% sodium chloride and added with guinea pig complement (Guinea Pig Serum and Saline Diluent, MP Biomedicals, Solon, Ohio) in a 1:1:1 ratio to the 2% RBC suspension. The complement is necessary to detect hemolyzing antibodies. Auto‐controls were performed using “donor” RBCs and serum. All tubes were incubated at 37°C for 30 minutes and then centrifuged for 1 minute at 1000*g*. The tubes were examined macroscopically for hemolysis and agglutination and microscopically for agglutination, using a predetermined scale of 0‐3+. Both agglutination and hemolytic reactions were used in the comparison to KIT. The semiquantitative agglutination score was 0 when no agglutination was observed; 1+ for weak agglutination with 2‐3 RBCs per agglutinate or transient RBC adherence; 2+ for moderate agglutination with only microscopic small agglutinins of 4‐10 RBCs per agglutinate; and 3+ for severe agglutination with any microscopic large agglutinins of >10 RBCs per agglutinate or any gross agglutination.

#### 2.3.2 Stall‐side gel crossmatch kit

**Figure 1:**
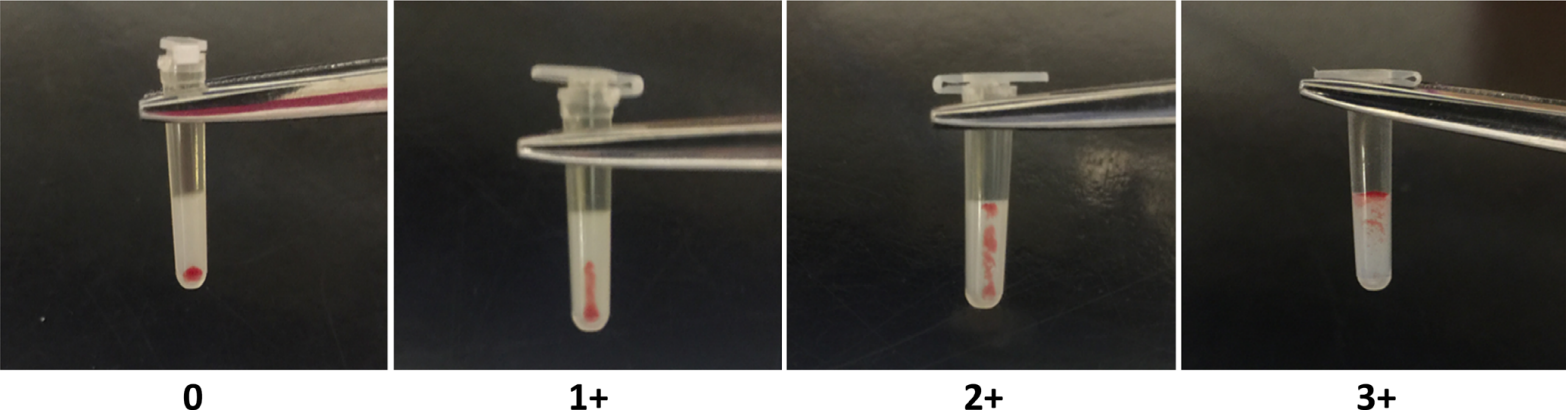
Scoring of agglutination in a crossmatch with the stall‐side kit (KIT). The degree of RBC retention in the gel is graded according to the following scale: 0, all RBCs at the bottom of the gel (compatible); 1+, few RBC agglutinates in the lower half of the gel but most RBCs at the bottom of the gel; 2+, RBC agglutinates dispersed throughout the gel, 3+, RBC agglutinates throughout gel and RBCs on upper surface. RBC retention of ≥1+ is considered incompatible

Crossmatches were performed using the gel matrix column KIT test according to the manufacturer's guidelines (Gel Test for Major Equine Crossmatch, Alvedia Veterinary Diagnostic Company, Limonest, France). “Donor” RBCs were allowed to settle by gravity for 5 minutes, the supernatant plasma removed and the “packed” RBCs were collected using the kit strip and then resuspended in the kit buffer, without washing the RBCs. Then, a 1:1 mixture of “donor” RBC suspension and “recipient” serum was added to a test tube. The mixture was lightly agitated by tapping using an index finger for approximately 10 seconds and then incubated at room temperature for 10 minutes. After incubation, the mixture was added to the top of the polypropylene gel column and centrifuged at 400 *g* for 5 minutes. The extent of RBC retention in the gel, corresponding to agglutination, was graded using a 0‐3+ scale (Figure 1). Hemolysis was not analyzed. To determine agreement among evaluators, results were archived by photography and were scored by 3 blinded independent evaluators.

### 2.4 Statistical analyses

#### 2.4.1 General approach

Results were categorized as yes/no for all tests except for unidentified anti‐RBC antibodies, which were considered as unknown (U). Data analysis was performed using JMP statistical software (v. 11.0, SAS Institute, Cary, North Carolina), except for sensitivity and specificity analysis, which was performed using MedCalc Statistical Software (v. 16.4.3, MedCalc Software bvba, Ostend, Belgium). Agreement (LAB versus KIT, photo evaluators of KIT reactions) beyond chance was calculated using Cohen's Kappa (κ) with confidence intervals (CI) and interpreted as no agreement (0.0‐0.20), fair (0.21‐0.40), moderate (0.41‐0.60), substantial (0.61‐0.80), and almost perfect (0.81‐1.00).^11^

#### 2.4.2 Aim 1

Sample size was a convenience sample size and was dependent on availability of 21 antibody and blood‐type screened horses and 3 anti‐sera. For this Aim, expected negative and positive reactions were determined based on the reference standard method; sensitivity and specificity with 95% CI were determined independently for LAB and KIT relative to expected reactions. Agreement between LAB and KIT, LAB and expected reaction, and KIT and expected reaction, respectively, were determined using Cohen's κ coefficient for dichotomous (yes/no) outcomes. Additionally, weighted κ for LAB versus KIT by strength of reaction (0 to 3+) was calculated.

#### 2.4.3 Aim 2

Formal sample size calculation was performed using the following assumptions: an expected prevalence of incompatible crossmatches in a convenience sample population consisting of primarily Thoroughbred (TB) and Warmblood (WB) horses to be 20%.^12^ We wanted to determine if the kit was able to detect at least 90% of incompatibilities detected by LAB with 95% CI of 80%‐100%. Sample size estimation determined 172 crossmatches as appropriate. Agreement between LAB and KIT was determined using Cohen's κ coefficient for yes/no outcomes and weighted κ for strength of reaction (0 to 3+) as described in Aim 1. Agreement between different evaluators for the KIT was determined accordingly. Agreement for crossmatch reactions in Aim 2 was stratified by recipient and donor gender (male versus female) and separately for TB and WB recipient and donor populations, given their largest representation in the sample set and the breed‐dependent prevalence of blood types. Assessment of the influence of recipient and donor age, respectively, on test agreement was explored using logistic regression with KIT as the dependent variable, LAB as independent variable, and LAB multiplied by age interaction as fixed effects. When the *P*‐value for the interaction <.05, data were further stratified and Cohen's κ calculated for strata.

## 3 RESULTS

The KIT took <20 minutes to perform, whereas the LAB method took 120‐180 minutes. Agreement of 3 independent photo evaluators with original evaluation of KIT was substantial with the 3 κ values ranging from 0.76 to 0.92. Cohen's κ between photo evaluators was almost perfect with the 3 κ values ranging from 0.83 to 0.98.

### 3.1 Aim 1

Within the group (Supplemental Table S1), there were horses with A, C, and Q RBC antigens, and serum with anti‐Aa, anti‐Ca, and anti‐Qab alloantibodies. Ninety crossmatches were performed using samples from 24 different horses, including 35 expected positive reactions, 36 expected negative reactions, and 19 reactions that were unknown (using recipients with unidentified anti‐RBC antibodies). Because of personnel availability, 2 KIT reactions were not performed, both expected negative reactions. The median age of tested horses was 17 years (range, 2‐22). Twenty‐one horses served as donors, providing RBCs for the reaction. They included 7 Thoroughbred (TB), 9 Warmblood (WB), 1 Quarter horse (QH), 1 American Paint, 1 Morgan, 1 Appaloosa, and 1 TB and Welsh pony mix breed. There were 11 mares, 7 geldings, and 3 stallions. The same 21 horses served as recipients and serum from an additional 3 horses (1 TB, 2 unknown breed, 3 mares) was used for recipients. Of the 21 donor horses, 18 (85.7%) had Aa, 20 (95.2%) had Ca, and 12 (57.1%) had Qabc blood types. Of the 24 recipients, 4 (16.7%) had anti‐Aa, 2 (8.3%) had anti‐Ca, 1 (4.2%) had anti‐Qab, and 9 (37.5%) had unidentified anti‐RBC antigen antibodies (Supplemental Table S1).

No LAB crossmatches had hemolysis without agglutination. The LAB had a sensitivity and specificity of 77.1% (59.9‐90.0 CI) and 77.8% (60.9‐89.9 CI), respectively. The KIT had a sensitivity and specificity of 91.4% (77.0‐98.2 CI) and 73.5% (55.6‐87.1 CI), respectively. The overall sensitivity and specificity of LAB and KIT crossmatch methods for expected positive and negative reactions were not significantly different, because the 95% CI overlapped. However, sensitivity for detecting incompatibilities with specific blood types was markedly different. The KIT was positive for all 19/19 (100%), whereas LAB was only positive in 11/19 (58%) expected anti‐Aa reactions. The LAB was positive for 10/10 (100%), whereas KIT was only positive for 7/10 (70%) expected anti‐Qab reactions. Both LAB and KIT were positive for 13/13 (100%) expected anti‐Ca reactions. When only unidentified anti‐RBC antibody was detected in recipients (n = 19), 17/19 (79%) and 16/19 (84%) of LAB and KIT reactions were negative, respectively. However, only 16 (84%, 1 positive and 15 negative) reactions with unidentified anti‐RBC antibodies were identical between LAB and KIT. Cohen's κ agreement was moderate (0.55, 0.36‐0.74) for LAB and substantial for KIT (0.65, 0.47‐0.82) versus expected reactions. Agreement was only fair (0.30, 0.08‐0.52) when comparing LAB with KIT for expected reactions. When comparing the strength of the reaction, the weighted κ for 69 expected positive and negative crossmatches was fair at 0.40 (0.23‐0.56); (Table 1). When unidentified anti‐RBC antibody reactions (n = 19) were included in the agreement assessment between LAB and KIT, κ for all agreements increased to 0.38 (0.19‐0.58). When the unidentified anti‐RBC antibody reactions were included for assessment of agreement based on strength of the reaction, weighted κ did not change (0.40, 0.23‐0.56).

**Table 1:** Agreement between LAB and KIT for 71 expected positive and negative crossmatches combined, separated by strength of reaction. Linear weighted κ = 0.40 (0.23‐0.56). This data does not include crossmatches using recipients with unidentified antibody (similar κ of 0.40)

		LAB			
KIT	**Reaction**	**0**	**1+**	**2+**	**3+**
	**0**	19	4	3	2
	**1+**	7	1	1	1
	**2+**	5	0	1	3
	**3+**	3	2	6	11

### 3.2 Aim 2

A total of 156 horses of unknown blood types and antibody profiles were recruited for reciprocal crossmatches, resulting in 156 crossmatches. Breeds included: 73 TB, 3 TB crosses, 31 WB, 10 Standardbred (SB), 9 QH, 7 draft horses, 5 Arabian, 1 Arabian cross, 2 Pony, 2 Paint, 4 Morgan, 2 miniature horses, 2 Appaloosa, and 5 other breeds (including mixed breed). There were 96 mares, 55 geldings, and 5 stallions. The median age was 11.5 years (range, 0.5‐31).

Only 1 crossmatch of the untyped horses in Aim 2 had moderate hemolysis without agglutination (LAB). Agreement between LAB and KIT for the 156 crossmatches performed in untyped horses was fair, with κ = 0.26 (0.11‐0.41). Weighted κ was also fair at 0.30 (0.17‐0.43) based on the strength of the reaction (Table 2).

**Table 2:** Agreement between LAB and KIT for 156 crossmatches in untyped horses, separated by strength of reaction. Linear weighted κ = 0.30 (0.17‐0.43)

		LAB			
KIT	**Reaction**	**0**	**1+**	**2+**	**3+**
	**0**	78	5	10	2
	**1+**	16	1	0	0
	**2+**	7	1	2	2
	**3+**	12	4	9	7

The influence of both donor and recipient breed was evaluated by stratification for TB and WB breeds, which contributed the largest number of horses. When TB recipients were evaluated separately, Cohen's κ between LAB and KIT increased to a moderate value of 0.52 (0.21‐0.84) and when WB recipients were evaluated separately, κ increased substantially to 0.69 (0.41‐0.97) when comparing LAB versus KIT. When TB donors were considered separately, agreement increased to a moderate κ of 0.49 (0.17‐0.80), but no increase in agreement was found for the WB donor population. Recipient and donor age had no influence on agreement (*P* ≥ .06) and neither recipient nor donor sex was associated with agreement (*P* ≥ .57).

## 4 DISCUSSION

Our objective was to evaluate the agreement between crossmatch tests done by LAB and KIT in horses with both known and unknown blood types. The KIT is a rapid, point‐of‐care test that can be performed in 20 minutes and could be used in a field setting for horses with life‐threatening anemia requiring prompt transfusion. We found that, when compared with the reference standard method, the agreement was moderate for LAB and substantial for KIT. Overall, LAB and KIT had similar sensitivity and specificity; however these differed by blood type. For horses of unknown blood types, the agreement between LAB and KIT was similarly fair, indicating that the preselection of reactions in Aim 1 did not bias the result substantially. Aim 1 of our study included serum from a horse with anti‐Qa antibodies, which, to our knowledge, has not been reported in recent studies, despite anti‐Qa antibodies being considered highly immunogenic and implicated in neonatal isoerythrolysis.^4^, ^9^ Aim 2 of our study was unique, where 156 individual horses of various breeds were crossmatched as donor‐recipient pairs and vice versa, leading to 156 crossmatches. This represents a larger data set compared to already existing studies, where a small number of horses were crossmatched multiple times.^6^, ^9^

### 4.1 Lack of a true reference standard

The blood typing and antibody screening results from the Hematology Laboratory at the University of California at Davis were considered as the reference standard with the understanding that this method is a similar type crossmatch method as used for LAB. However, there is currently no true gold standard method to better evaluate sensitivity and specificity of an equine crossmatch. Additionally, even if reactions are predicted by blood type, the actual occurrence of transfusion reactions may differ and may be caused by other factors not measured with a crossmatch test, such as leukocytes, platelets, proteins, or poorly documented RBC antigens.^1^, ^4^ Therefore, the true gold standard would be to actually transfuse blood between 2 horses after the crossmatch to determine compatibility and clinical relevance of these tests. Doing so was beyond the scope of our study.

### 4.2 Differences between LAB and KIT methods

The LAB procedure uses the standard tube agglutination method. This method requires a certain level of expertise and training for accurate interpretation of results, particularly in distinguishing weak positive reactions. The method of pellet agitation and the subjective nature of the grading could affect the interpretation of results.^13^ Subjectivity was minimized in our study by using additional observers to confirm the results. The KIT uses the gel column method. The gel column itself is easy to read, with the interpretation of the results essentially being independent of the skill of the reader,^14^ as evidenced by substantial to near perfect agreement among observers for this method.

The LAB method evaluates 2 more aspects of incompatibility than does KIT. The LAB detects both macroscopic and microscopic agglutination, compared to KIT, which only examines agglutination macroscopically. Additionally, LAB detects hemolysis and KIT is not designed to do so. These differences might be expected to result in lower sensitivity for KIT, but we did not observe differences. The gel likely captures microscopic agglutination even though it is evaluated macroscopically. Additionally, it appears that anti‐RBC antibodies that cause hemolysis alone without concurrent agglutination are quite rare, and the inability of the kit to detect hemolysis likely will not markedly affect the sensitivity of the test.^6^ Yet, this still remains a limitation of KIT.

Another difference between methods is that LAB dilutes the recipient's serum at 1:2 and KIT does not predilute serum. Additionally, with the LAB method, complement is added to the suspension, further adding to the dilutional effect, which might also explain the numerically lower sensitivity of the LAB method for expected positive reactions in Aim 1 (the reference method used less dilute serum).

### 4.3 Discrepancies between tests

The dilutional effect could contribute to discrepancies between the LAB and KIT results for the Aa blood group expected positive reactions (ie, LAB had a higher rate of false‐negative reactions than did KIT). Anti‐Aa antibody‐positive recipient serum was available from 2 horses for our study and false‐negative reactions for LAB were seen with both antisera. Because numerical values are not reported for titers, it is possible that the anti‐Aa antibody titers were low in these 2 horses and, combined with the predilution of serum, resulted in a false negative with the LAB method. In contrast, the incompatibility was still detectable with the undiluted KIT method. To better interpret these results and understand the clinical risk associated with these false‐negative reactions, future studies should include repeating the discrepant crossmatches with minimal dilution of serum in the LAB procedure and performing trial transfusions as described above. Conversely, there were false negatives with anti‐Qab reactions with KIT. The reason for these false negatives is unknown, but this is a limitation of the kit and additional testing of other anti‐Qab sera would be worthwhile.

A possible explanation for false‐negative test results for both methods is transient antibody production, such that antibodies were no longer detectable in horses when performance of the crossmatch procedures lagged behind screening. Additionally, instability of antibodies with frozen storage of serum, or different levels of expression of antigen on the surface of the donor RBCs used in the reference method versus the 2 methods used here are possible reasons to consider. Conversely, antibodies newly acquired after screening may result in false‐positive reactions. A recent crossmatching study showed unexpected changes in antibody detection over several years in a group of horses.^9^ The time from blood typing and antibody screening of horses in Aim 1 to crossmatching ranged from a minimum of 5 months to a maximum of 16 months. Therefore, the discrepancy between screenings could be due to a true change in the presence or absence of antibodies, a change in the capacity of the test to detect antibodies in low concentrations or weak immunoreactivity.^9^ The latter study found that most of the discrepancies between tests were associated with horses that had changes in their antibody profile.^9^ Similarly, the repeatability of blood typing with the reference method is not known and it is possible that some of the donors were incorrectly blood typed (particularly because weak agglutinins may be missed when microscopic methods are not used to assess for agglutination). Future studies could include repeating the antibody screening and blood typing to better understand differences in performance of the methods.

### 4.4 Clinical implications of breed on test performance

Expected performance of both LAB and KIT will depend on the prevalence of blood types within the tested horse population. In horses, blood factor frequencies are breed‐dependent. For example, 85% of TBs are Qa positive, in comparison to 99% of Morgans and Standardbreds that are Qa negative.^12^ Therefore, there is a high likelihood of causing a transfusion reaction if a Qa‐positive TB was used as a donor for a Morgan or Standardbred with RBCs lacking the Qa antigen (presuming they have preexisting anti‐Qa antibodies). For anti‐Aa antibodies, the LAB method had a higher rate of false‐negative reactions, but for anti‐Qab antibodies, KIT had a higher rate of false‐negative reactions. The KIT would perform well in a population in which a large proportion of recipients is Aa negative and Qa positive (eg, QH are 26% Aa− and 32% Qa+). However, it would not perform as well for a population of Standardbreds, which are 99% Qa negative, especially if a TB donor was used (85% Qa+), because there is a higher likelihood of a false‐negative result for Qa incompatibility when using KIT.^12^ A false‐negative test result could result in missing a potentially clinically relevant transfusion reaction.

In our population, there was greater agreement between LAB and KIT when TB and WB were recipients or TB were donors, than for the mixed general population. This is likely because KIT and LAB perform best in a population with a high frequency of Aa and Qa blood types (and lower frequency of anti‐Aa or anti‐Qa antibodies), such as TB horses (100% Aa positive and 86% Qa positive in 7 tested horses (Supplemental Table S1).^12^ The frequency of blood types in WB has not been published, but 78% of the 9 tested WB in Aim 1 were Aa positive and 11% were Qa positive (Supplemental Table S1).

### 4.5 Clinical relevance

As previously mentioned, weak antibody titers could have contributed to discrepant test results. However, the actual clinical relevance of these weak titers is unknown.

Based on studies in foals with neonatal isoerythrolysis, anti‐Ca antibodies are reported to not be as clinically relevant as anti‐Aa and anti‐Qa antibodies.^15^ A previous study also showed that 3+ incompatible crossmatches with anti‐Ca antibodies only predicted mild febrile and tachycardic transfusion reactions that did not prevent completing the transfusion.^4^ Thus, the severity of the crossmatch incompatibility with anti‐Ca reactions does not necessarily correspond to the clinical response in a patient. Whether the extent of crossmatch incompatibility correlates with reaction severity with other blood types is not known. Similarly, the clinical relevance of reactions against unidentified RBC antigens is unknown. Neither LAB nor KIT had perfect sensitivity for expected reactions, which could mean that both are unreliable in detecting every possible incompatibility. Horses undergoing transfusion should be monitored closely, even for predicted compatible transfusions, because of possible false‐negative results and because some reactions appear to occur secondary to leukocytes, platelets, proteins, or poorly described RBC antigens that are not detected by current compatibility testing.^4^, ^9^

### 4.6 Limitations

All KIT crossmatches were performed by the same individual, which optimized its performance. Several different personnel performed the LAB crossmatches according to staffing schedules. This mimics what would occur in clinical practice, but it could have introduced some additional variability to the results. We only had a limited number of horses with antibodies against known RBC antigens, restricting expected positive reactions to a set number of recipients. For example, we only had 1 antiserum against the Qab antigen. Because the expected performance of both methods appears to depend on the prevalence of blood types within the tested horse population, another limitation is that breed distribution was restricted in this sample set and KIT performance ideally should be assessed in the patient population in which it would be used.

### 4.7 Conclusion

Agreement with expected reactions between KIT and LAB was substantial and moderate, respectively, but only fair when comparing both methods with each other in both blood typed and untyped mixed populations of horses. Agreement was blood type dependent and improved when stratifying data by the 2 most represented breeds (TB and WB). Thus, we conclude that the performance of both methods will depend on the prevalence of blood types within the tested horse population. Future studies aimed at identifying the reasons for discrepant method results, having a clearer understanding of how anti‐RBC antibody profiles change over time, and determining if positive crossmatch reactions correlate with in vivo transfusion reactions are needed.

#### CONFLICT OF INTEREST DECLARATION

Authors declare no conflict of interest.

#### OFF‐LABEL ANTIMICROBIAL DECLARATION

Authors declare no off‐label use of antimicrobials.

#### INSTITUTIONAL ANIMAL CARE AND USE COMMITTEE (IACUC) OR OTHER APPROVAL DECLARATION

Cornell University IACUC Approval for blood sampling in horses.

#### HUMAN ETHICS APPROVAL DECLARATION

Authors declare human ethics approval was not needed for this study.

## Supporting information


**Appendix** S1: Supporting Information
