# Supplementary material for: Agreement of stall‐side and laboratory major crossmatch tests with the reference standard method in horses
Source: J Vet Intern Med. 2020 Feb 4;34(2):941–8. doi: 10.1111/jvim.15710 (PMC7096648; doi:10.1111/jvim.15710)
Supplement: Supplementary file 1 — Appendix S1: Supporting Information [file JVIM-34-941-s001.pdf]

Supplementary Table 1. Signalment, blood types and antibody profiles of horses in Aim 1 along with the number of times each was a donor or recipient.

Horse	Serum antibodies Lysins	Serum antibodies Agglutinins	Blood Type	Breed	Sex	Age (years)	Cross-matched as donor	Cross-matched as recipient
1	Qab	Qab	ND	unknown	M	unknown	0	15
2	Neg	Aa, anti-donkey	Ca	Paint	M	14	9	8
3	Neg	Neg	Aa Ca Qabc	QH	M	8	7	8
4	Ca	Aa Ca	Ab Ka Pa Qc	WB	G	16	8	7
5	Ca	Ca	Aa Qabc	TB	M	3	0	6
6	Neg	Neg	Aa Ca Pa Qabc	TB	M	17	7	6
7	Neg	Neg	Aab Ca Pb Qabc	TB	M	21	2	5
8	Neg	Neg	Aa Ca Qabc	TB	M	17	7	5
9	Neg	UnID	Aa Ca Pa	WB	S	22	8	4
10	Neg	Neg	Aab Ca Pa Ua	WB	S	14	7	4
11	UnID	Aa, anti-donkey	ND	unknown	M	22	0	3
12	Neg	UnID	Aabc,Ca, Pab, Qabc	WB	S	18	8	3
13	Neg	Aa, anti-donkey	Ab Ca, Pa, Ua	WB	M	21	4	2
14	Neg	UnID	Aa Ca Pb Qabc	TB	G	18	3	2

15	Neg	UnID, anti-donkey	Aa Ca Qabc	TB	G	10	2	2
16	Neg	Neg	Aa Ca Pa Qabc Ua	WB	M	19	2	2
17	Neg	Neg	Aa Ca Pa Qb	Morgan	M	2	2	1
18	Anti-donkey	Anti-donkey	Aa Ca Pa Qabc Ua	WB	G	17	2	1
19	Neg	Neg	Aa Ca Ka	WB	M	17	3	1
20	Neg	Neg	Aa Ca Qabc	TB	G	7	2	1
21	Neg	UnID	Aa Ca Pa Qabc	Appaloosa	G	15	2	1
22	Neg	UnID	Aa Ca	TB x Welsh	M	14	2	1
23	Neg	Neg	Aa Ca	TB	G	14	2	1
24	Neg	UnID, anti-donkey	Aa Ca Ua	WB	M	17	1	1

Neg = negative; UnID = unidentified antibody; QH = quarter horse; WB = warmblood; TB = thoroughbred; M = mare; G = gelding; S = stallion
